# Sex-related gene expression profiles in the adrenal cortex in the mature rat: Microarray analysis with emphasis on genes involved in steroidogenesis

**DOI:** 10.3892/ijmm.2015.2064

**Published:** 2015-01-09

**Authors:** MARCIN TREJTER, ANNA HOCHOL, MARIANNA TYCZEWSKA, AGNIESZKA ZIOLKOWSKA, KAROL JOPEK, MARTA SZYSZKA, LUDWIK K MALENDOWICZ, MARCIN RUCINSKI

**Affiliations:** Department of Histology and Embryology, Poznan University of Medical Sciences, Poznan, Poland

**Keywords:** rat, adrenal cortex, transcriptome, gene profiling, functional annotation clustering, sex differences

## Abstract

Notable sex-related differences exist in mammalian adrenal cortex structure and function. In adult rats, the adrenal weight and the average volume of zona fasciculata cells of females are larger and secrete greater amounts of corticosterone than those of males. The molecular bases of these sex-related differences are poorly understood. In this study, to explore the molecular background of these differences, we defined zone- and sex-specific transcripts in adult male and female (estrous cycle phase) rats. Twelve-week-old rats of both genders were used and samples were taken from the zona glomerulosa (ZG) and zona fasciculata/reticularis (ZF/R) zones. Transcriptome identification was carried out using the Affymetrix^®^ Rat Gene 1.1 ST Array. The microarray data were compared by fold change with significance according to moderated t-statistics. Subsequently, we performed functional annotation clustering using the Gene Ontology (GO) and Database for Annotation, Visualization and Integrated Discovery (DAVID). In the first step, we explored differentially expressed transcripts in the adrenal ZG and ZF/R. The number of differentially expressed transcripts was notably higher in the female than in the male rats (702 vs. 571). The differentially expressed genes which were significantly enriched included genes involved in steroid hormone metabolism, and their expression levels in the ZF/R of adult female rats were significantly higher compared with those in the male rats. In the female ZF/R, when compared with that of the males, prevailing numbers of genes linked to cell fraction, oxidation/reduction processes, response to nutrients and to extracellular stimuli or steroid hormone stimuli were downregulated. The microarray data for key genes involved directly in steroidogenesis were confirmed by qPCR. Thus, when compared with that of the males, in the female ZF/R, higher expression levels of genes involved directly in steroid hormone synthesis were accompanied by lower expression levels of genes regulating basal cell functions.

## Introduction

It is well known that adult female rats have heavier adrenal glands than males of the same age. This difference appears only after puberty and is dependent upon sex hormones. Earlier, mainly morphologic, cytological and gravimetric data on this subject had been extensively reviewed by Bachmann in 1954 (1). In the rat, adrenal sex dimorphism is accompanied by functional differences, with females secreting greater amounts of corticosterone than males (reviewed in refs. 2–5). Furthermore, female rats show greater adrenocorticotropic hormone (ACTH) and corticosterone responses to stress and these hormonal responses are modified by gonadectomy and gonadal hormone replacement (6–8). Numerous studies have suggested that observed sex differences in the rat adrenal structure and function are dependent on the inhibitory effects of testosterone on the hypothalamo-pituitary-adrenal (HPA) axis, while estrogens exert opposite effects (reviewed in refs. 4–5,9–12). However, the molecular bases of the above outlined sex-related differences in rat adrenal cortex structure and function have not yet been fully elucidated.

The introduction of gene expression microarray technology opens the possibility of discovering genes that may contribute to various biological effects. As regards the adrenal gland, an example of such a study is the identification of genome-wide changes in gene expression following the treatment of Y1 mouse adrenocortical cells with ACTH (13). In this cell line, ACTH affected the levels of 1,275 different transcripts, and only 133 were previously known as corticotropin-affected. Moreover, the authors demonstrated that the cAMP/protein kinase A and protein kinase C pathways appeared to account for approximately 60% of the effects of ACTH (13). Furthermore, by means of laser-capture microdissection, Nishimoto *et al* (14,15) identified hundreds of transcripts with differential expression in the zona glomerulosa (ZG) and zona fasciculata (ZF) of adult male rats.

As far as sex-related differences in the adrenal cortex are concerned, recently, El Wakil *et al* performed a genomic analysis of gene expression in the mouse adrenal gland (16). In their data, not considering the transcription factors, nuclear receptor subfamily 5, group A, member 1 (Nr5a1) and nuclear receptor subfamily 0, group B, member 1 (Nr0b1), none of the genes directly involved in steroid hormone biosynthesis showed a differential expression in the male and female mouse adrenal glands (16).

Therefore, in the present study, using the adrenal glands of mature male and female rats, we performed whole transcriptome analyses that allowed us to compare the expression levels of approximately 27,000 genes by applying microarray technology. Gene expression analyses were performed separately on ZG and ZF/reticularis (ZF/R) samples. In the ZG, we revealed 32 differentially expressed genes, while 233 such genes were found in the ZF/R. The functional profiles of the differentially (male/female) expressed genes were characterized by means of the GeneAnswers package of Bioconductor or by the Database for Annotation, Visualization and Integrated Discovery (DAVID) tool, as previously described (17,18). Both methods revealed functional profiles of the differentially (male/female) expressed genes and their role in the rat adrenal gland was analyzed.

## Materials and methods

### Animals

Adult female and male Wistar rats (12 weeks old; final body weight, 120–150 g) were obtained from the Laboratory Animals Breeding Center, Department of Toxicology, Poznan University of Medical Sciences, Poznan, Poland. The animals were maintained under standardized conditions of light (14:10 h light-dark cycle, illumination onset 06.00) at 23°C with free access to standard food pellets and tap water. Female rats were used in the estrous cycle phase, which was determined according to the cell types observed in the vaginal smear. Following decapitation (between 09:00 and 10:00), the adrenal glands were promptly removed, freed of adherent fat and processed for analysis. Briefly, under a stereomicroscope, the male and female (both groups, n=6) rat adrenal glands were decapsulated to separate the ZG from the ZF/R. The medulla of the adrenal gland was removed and was not used in our analysis. The Local Ethics Committee for Animal Studies at the Poznan University of Medical Sciences approved the study protocol. Unless otherwise stated, all reagents were obtained from Sigma-Aldrich (St. Louis, MO, USA) or from Avantor Performance Materials Poland S.A. (Gliwice, Poland).

### RNA isolation

RNA isolation from samples of adrenal glands was carried out as previously described (19–23). Briefly, we used TRI reagent (Sigma-Aldrich) and the isolated RNA was purified on columns (RNeasy mini kit; Qiagen, Hilden, Germany). The quantity of the total RNA was determined spectrophotometrically (optical density at 260 nm) and its purity was estimated by a 260/280 nm absorption ratio (>1.8) (NanoDrop spectrophotometer; Thermo Scientific, Waltham, MA, USA). RNA integrity and quality were examined using the Bioanalyzer 2100 (Agilent Technologies, Inc., Santa Clara, CA, USA). Evaluated RNA Integrity Numbers (RINs) were between 8.5 and 10 with the average of 9.2. The RNA concentration in each sample was diluted to 100 ng/*μ*l with an OD260/OD280 ratio of 1.8/2.0.

### Microarray analysis

**Figure 1:**
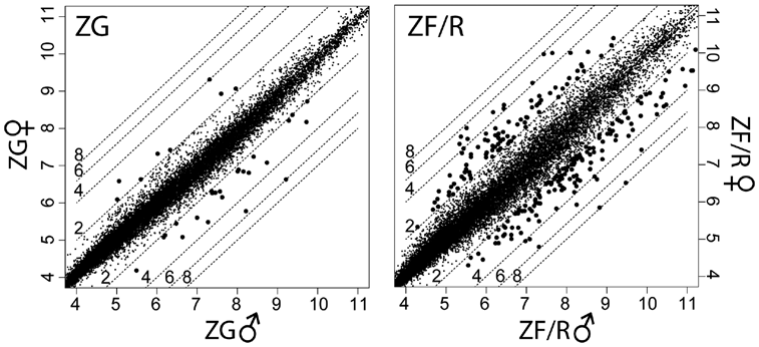
Scatter plot graphs of gender-specific differences in gene expression in the ZG and ZF/R of adrenal glands of adult female rats, in comparison to adult male rats (Affymetrix^®^ Rat Gene 1.1 ST Array). Dotted lines indicate cut-off values (2,4,6,8 fold change in expression). The left upper part of the graphs indicates higher expression levels in the female compared to the male adrenal glands; the right lower part of the graph indicates lower expression levels in the female compared to the male adrenal glands (differences in expression ≥2; p<0.05). Names of genes are not shown. ZG, zona glomerulosa; ZF/R, zona fasciculata/reticularis.

Microarray analysis was carried out as previously described (21–23). Isolated total RNA (100 ng) was mixed with 1.5 *μ*l of Poly-A RNA control solution and subjected to reverse transcription. The obtained cDNA was used for *in vitro* transcription to prepare antisense RNA (aRNA) by incubation at 40°C for 16 h. Following purification, the aRNA was applied for the second round of sense cDNA synthesis using the WT Expression kit (Ambion, Austin, TX, USA). All subsequent steps were performed using the Affimetrix microarray system (Affymetrix, Santa Clara, CA, USA). The obtained cDNA was used for biotin labeling and fragmentation by Affymetrix GeneChip^®^ WT Terminal Labeling and Hybridization. Biotin-labeled fragments of cDNA (5.5 *μ*g) were hybridized to the Affymetrix^®^ Rat Gene 1.1 ST Array Strip (45°C/24 h). Each array comprised of >720,000 unique 25-mer oligonucleotide probes, which included >27,000 genes. Up to 25 unique probes sequences were hybridized to a single transcript. Following hybridization, each array strip was washed and stained using the Fluidics Station of GeneAtlas system (Affymetrix). The array strips were scanned using the Imaging Station of the GeneAtlas system. A preliminary analysis of the scanned chips was performed using Affymetrix GeneAtlas™ Operating Software. The quality of gene expression data was examined according to the quality control criteria provided with the software. The intensity of fluorescence was converted to numerical data by generating CEL files. The obtained CEL files were imported into downstream data analysis software. Unless otherwise stated, all presented analyses and graphs were performed using Bioconductor and R programming language, as previously described (24). Each CEL file was merged with a description file (downloaded from the Affymetrix webpage). In order to perform background correction, normalization and summarization of the results, we used the Robust Multiarray Averaging (RMA) method. The statistical significance of the analyzed genes was examined by moderated t-statistics from the empirical Bayes method. The obtained p-values were corrected for multiple comparisons using the Benjamini and Hochberg’s false discovery rate (statistical method incorporated into Bioconductor calculations) (25). The selection of genes with a significant change in expression was based on a p-value <0.05 and an expression fold change ≥2. Fold change calculations were performed either for appropriate zones of male and female rats or for the ZG and ZF/R of adrenal glands in both genders (Fig. 1).

### Functional analysis by GeneAnswer

Singular enrichment analysis (SEA) was performed as previously described (15,26,27). Selected sets of differentially expressed genes were applied to functional analysis using the GeneAnswers package of Bioconductor which, among other, allows us to interpret a list of genes in the context of their participation in particular biological processes (GO.BP) (28). Gene ontology analyses were also performed using the web deposited manual (http://www.bioconductor.org/packages/release/bioc/vignettes/GeneAnswers/inst/doc/geneAnswers.pdf). Lists of differentially expressed genes were combined as tables and were subjected to further analyses. Since our dataset comprised 2 comparison sets (male vs. female ZG and male vs. female ZF/R) we performed multigrup gene analyses. The GeneAnswers package allowed to test the enrichment of each GO.BP category in a gene list using a well-defined hypergeometric statistical test. The p-value was determined based on the number of genes differentially expressed in the investigated GO category. This test was performed separately for the ZG and ZF/R and the results are presented in the respective figures.

### Functional analysis by DAVID

The other analysis was performed by functional annotation clustering using DAVID. This database provides functional annotation tools for understanding the biological meaning behind a large list of genes (www.david.abcc.ncifcrf.gov). Among the many functions, DAVID allows us to discover enriched function-related gene groups and to cluster similar annotation terms (17,18).

All procedures were performed according to the web provided manual (http://david.abcc.ncifcrf.gov/content.jsp?file=functional_annotation.html). The official symbols of genes which were differentially expressed (both in male and female ZG and ZF/R) were loaded separately into DAVID as a gene list. Annotations and background (total number of genes in the rat) were limited only to *Rattus norvegicus.* In order to obtain the most meaningful clusters, the threshold of EASE score (a modified Fisher exact p-Value) for gene enrichment analysis was set ≤0.05. Again, the obtained p-values were corrected for multiple comparisons using the Benjamini and Hochberg’s false discovery rate (25). Analyses were performed separately for the ZG and ZF/R zones of the adult male and female rats, as indicated in the descriptions provided with the figures and tables.

### Validation by RT-qPCR

The methods applied for RT-qPCR were as described in previous studies (19–23). Reverse transcription was performed using AMV reverse transcriptase (Promega Corp., Madison, WI, USA) with Oligo(dT) (PE Biosystems, Warrington, UK) as primers in the temperature of 42°C for 60 min (Thermocycler UNO II; Biometra, Goettingen, Germany). The primers used were designed by Primer 3 software (Whitehead Institute for Biomedical Research, Cambridge, UK) (Table I). The primers were purchased from the Laboratory of DNA Sequencing and Oligonucleotide Synthesis, Institute of Biochemistry and Biophysics, Polish Academy of Sciences, Warsaw, Poland. qPCR was performed using the Lightcycler 2.0 instrument (Roche Diagnostics Corp., Indianapolis, IN, USA) with the 4.05 software version. Using the above-mentioned primers, the SYBR-Green detection system was applied. Each 20 *μ*l reaction mixture contained 4 *μ*l template cDNA (standard or control), 0.5 *μ*M of each gene-specific primer and a previously determined optimum MgCl_2_ concentration (3.5 *μ*M for one reaction). LightCycler FastStart DNA Master SYBR-Green I mix (Roche Diagnostics Corp.) was used. The real-time PCR program included a 10-min denaturation step to activate the TaqDNA polymerase, followed by a 3-step amplification program: denaturation at 95°C for 10 sec, annealing at 56°C for 5 sec, and extension at 72°C for 10 sec. The specificity of the reaction products was examined by the determination of the melting points (0.1°C/sec transition rate).

### Statistical analysis

The RT-qPCR data are expressed as the means ± SE, and the statistical significance of the differences between the control and experimental groups was estimated using the Student’s t-test. A p-value <0.05 was considered to indicate a statistically signficiant difference.

## Results

**Figure 2:**
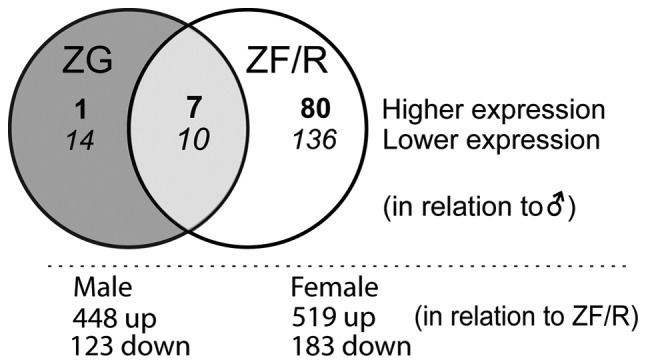
Venn diagram of differentially expressed genes in the ZG and ZF/R of adrenal glands of adult female rats, in comparison to adult male rats. Middle section shows the number of genes which had similar expression patterns in both zones. Bold font indicates higher expression levels; italic font indicates lower expression levels. In the lower part of figure, the numbers of genes differentially expressed in the ZG, in comarison to the ZF/R are shown (separately for adrenal glands of adult male and female rats. ZG, zona glomerulosa; ZF/R, zona fasciculata/reticularis).

To obtain more precise data on sexually dimorphic gene expression in the adrenal glands of adult male and female rats, we performed analyses of the samples of the ZG and ZF/R of the glands. Due to the nature of the applied experiment, all data were analyzed in relation to the adrenal glands of the male rats. The mean expression value of each gene was presented in scatter plot graphs (Fig. 1). The left upper part of the graphs shows genes, the expression levels of which were higher in the female than in the male adrenal glands. In the right lower part of the graphs, genes are also shown, the expression levels of which were lower in the female than in the male adrenal glands. By applying the previously mentioned cut-off parameters (fold change ±2; p<0.05), the Affymetrix Rat Gene 1.1 ST Array data revealed 32 differentially expressed genes in the ZG, and 233 genes in the ZF/R. A Venn diagram demonstrated their localization to the adrenocortical zones examined (Fig. 2). Of these, in the ZG only 15, while in the ZF/R 216 genes were exclusively expressed; 17 genes were regulated both in the ZG and ZF/R. In the ZG, the expression levels of 24 genes were lower and 8 were higher in the female rats. More pronounced sex-related differences were observed in the ZF/R. In this compartment of the rat adrenal cortex, the expression levels of 146 genes were lower and those of 87 genes were higher in the female rats. In both the ZG and ZF/R, the expression levels of 10 genes were lower and 7 were higher in the female rats.

We also identified transcripts with differential expression in the ZG and ZF/R of adult male and female rats (Fig. 2). In male rats, compared to the ZF/R, in the ZG 448 genes were upregulated and 123 downregulated. In females, these figures were 519 and 183, respectively.

**Figure 3:**
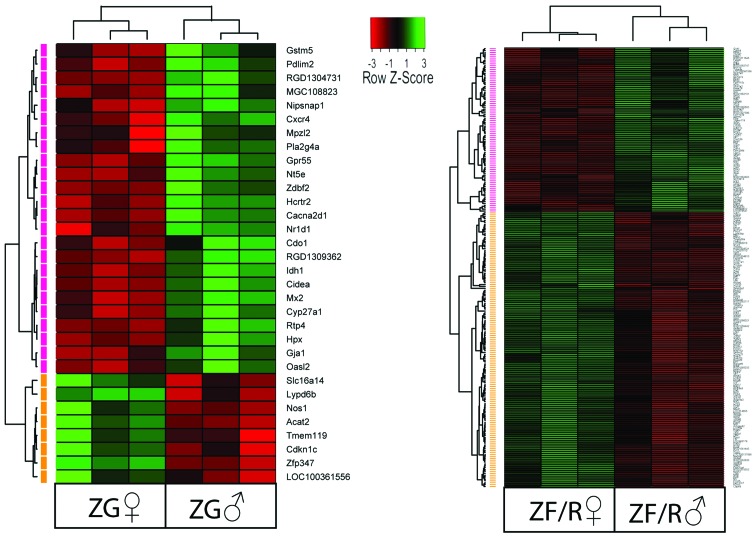
Heat map representation of microarray analysis for the gender-specific differentially expressed transcripts within the ZG and ZF/R of adult male and female rats. Arbitrary signal intensity acquired from microarray analysis is represented by colors. Studied genes were clustered by means of hierarchical clustering algorithm (green, higher; red, lower expression, in comparison to male gland). Hierarchical clustering was performed on log2 signal intensity data. These values were resized to Row Z-Score scale for any single genes (from -3, the lowest expression to +3, the highest expression). ZG, zona glomerulosa; ZF/R, zona fasciculata/reticularis.

Each of the raw expression values from gender-specific genes was grouped using a hierarchical clustering algorithm. The results of this analysis are presented as a heat map (Fig. 3). The clustering confirmed that in the ZG, the expression levels of 24 genes were lower and 8 were higher in the female rats and the symbols of these genes are shown. The same applied to the ZF/R; however, in this compartment, the expression levels of 146 genes were lower and those of 87 genes were higher in the female rats.

**Figure 4:**
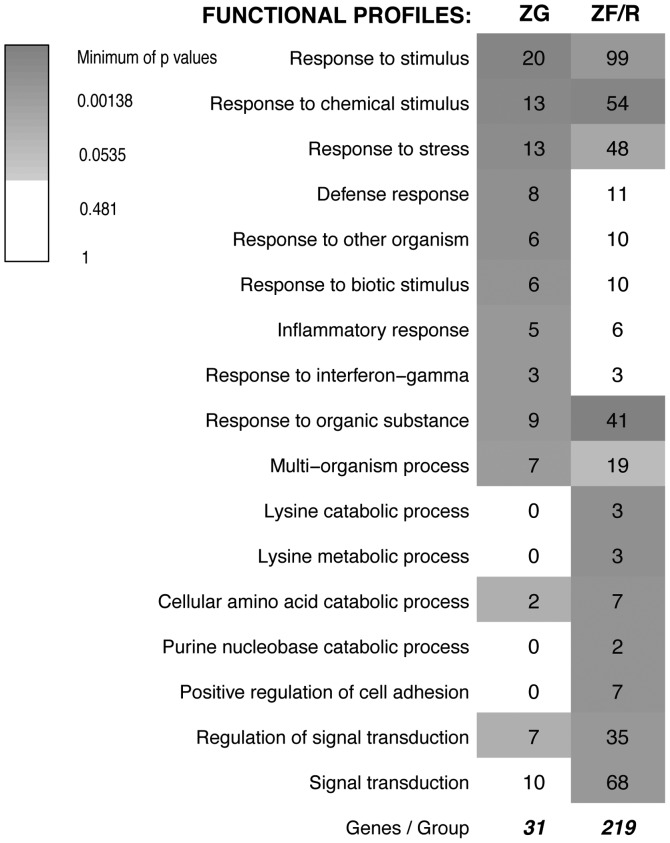
Functional profiles of differentially expressed genes determine by the GeneAnswers package (as of December 16, 2013), based on the Gene Ontology. Biological Process (GO.BP) database. Presented numbers show quantity of genes (identified in the present study) which are involved in described biological processes (functional profiles). Genes were analyzed from both male and female adrenal glands. Grey background marks functional groups of genes with statistically significant differences (hypergeometric test, p<0.05). ZG, zona glomerulosa; ZF/R, zona fasciculata/reticularis.

Subsequently, we performed SEA followed by functional analysis using the GeneAnswers package of Bioconductor which, among other things, allows us to interpret a list of genes in the context of their participation in a certain particular biological process (GO.BP). The original results of GeneAnswer analysis (as of December 16, 2013) revealed 17 groups of genes with different functional profiles. These profiles were rather general (for example ‘response to stimulus’, ‘response to chemical stimulus’, ‘response to organic substances’) and the prevailing number of the obtained profiles were out of our interest (Fig. 4). It should be emphasized that the GO database is composed of some general, as well as specific categories with a similar meaning and, therefore, a single gene may be mapped to several GO terms and may be counted more than once. Moreover, GO functional annotations of genes are still in the developing stage and are far from complete. Since this analysis yielded rather unsatisfactory results, we decided to analyze our results using the DAVID system.

The DAVID system is a powerful tool that allows us to discover enriched functionally related gene groups and to cluster similar annotation terms. This system extracts data from numerous databases and, as evidenced by the Science Citation Index, this system is gaining wide popularity among molecular biologists. We performed separate analyses for the ZG and ZF/R. For the ZG, with the selected cut-off value (p<0.05), 3 annotation clusters were obtained (ordered by enrichment score) (Table II). On the other hand, for the ZF/R 5 clusters were obtained. All clusters (ZG and ZF/R) combined 516 genes (counts). This figure indicated that numerous genes participated in more than one annotation cluster. In the ZG 90 counts were either up- or downregulated, while in the ZF/R the number of counts was notably higher, i.e., 426.

For the ZG, annotation clusters 1 and 2 combined genes involved in the regulation of ion transport, while cluster 3 contained transcripts linked to response to hormones (Table II). In the case of ZF/R, we obtained 5 clusters. The first cluster combined genes regulating steroid biosynthesis and metabolism. Cluster 2 incorporated genes primarily connected with cell fractions, while annotation cluster 3 (with 13 subclusters) combined transcripts primarily connected with oxidation/reduction processes. Cluster 4 was composed of genes regulating responses to nutrients and to extracellular stimuli or steroid hormone stimuli. Annotation cluster 5 also contained genes involved in response to stimuli, among which were ‘response to hormone stimulus’ (subcluster 2) and ‘response to peptide hormone stimulus’ (subcluster 7).

**Figure 5:**
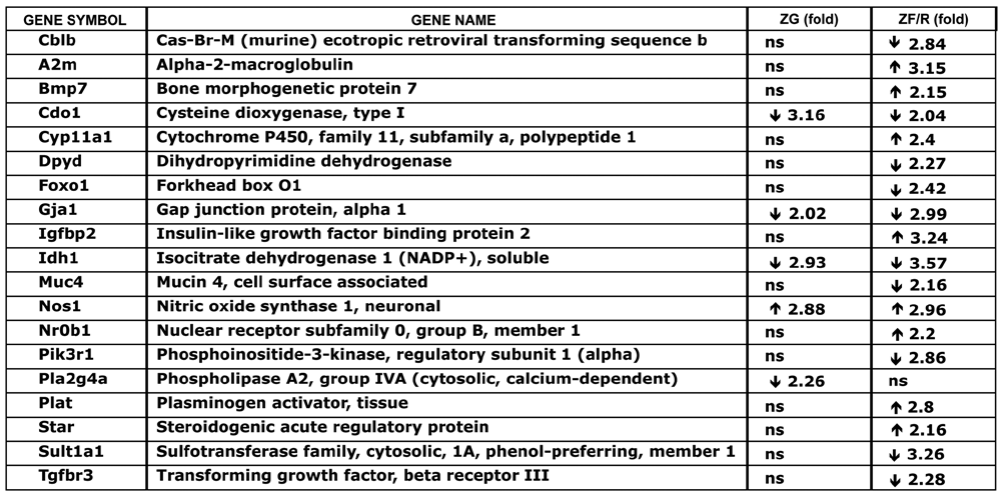
List of genes represented in cluster 3, ‘response to hormone stimulus’ (ZG) and cluster 5, subcluster ‘response to hormone stimulus’ (ZF/R) (shown in Table II, DAVID analysis) which are differentially expressed in the ZG and ZF/R of adrenal glands of adult female rats, in comparison to adult male rats. Downward-facing arrowhead indicates a lower expression in females than in males; upward-facing arrowhead indicates a higher expression in females than in males; ns, differences statistically not significant; ZG, zona glomerulosa; ZF/R, zona fasciculata/reticularis.

Subsequently, we analyzed a list of genes which were differentially expressed in the ZG and ZF/R of the adrenal glands in adult female rats, in comparison to adult male rats and annotated as a ‘response to hormone stimulus’ (for the ZG from cluster 3, and for ZF/R from cluster 5 shown in Table II). Of these genes, the expression levels of cysteine dioxygenase type 1 (Cdo1), gap junction protein, alpha 1 (Gja1), isocitrate dehydrogenase 1 (Idh1) and phospholipase A2, group IVA (Pla2g4a) were lower and those of nitric oxide synthase 1 (Nos1) were higher in the ZG of female rats, when compared with the males (Fig. 5). More pronounced differences were observed in the ZF/R zones of the rat adrenal cortex. Of the 19 genes forming the above-mentioned subcluster, the expression levels of 7 of these genes were higher and those of 10 genes were lower in the female adrenal glands. In the female ZF/R, higher expression levels were observed in the following genes: alpha-2-macroglobulin (A2m), bone morphogenetic protein 7 (Bmp7), cytochrome P450, family 11, subfamily A, polypeptide 1 (Cyp11a1), insulin-like growth factor binding protein 2 (Igfbp2), Nos1, Nr0b1 and plasminogen activator, tissue (Plat). On the contrary, in the female ZF/R of the adrenal cortex, lower expression levels were observed in the following genes: Cbl proto-oncogene B, E3 ubiquitin protein ligase (Cblb), Cdo1, dihydropyrimidine dehydrogenase (Dpyd), forkhead box O1 (Foxo1), Gja1, Idh1, mucin 4, cell surface associated (Muc4), phosphoinositide-3-kinase, regulatory subunit 1 (alpha) (Pik3r1), sulfotransferase family, cytosolic, 1A, phenol-preferring, member 1 (Sult1a1) and transforming growth factor, beta receptor III (Tgfbr3).

**Figure 6:**
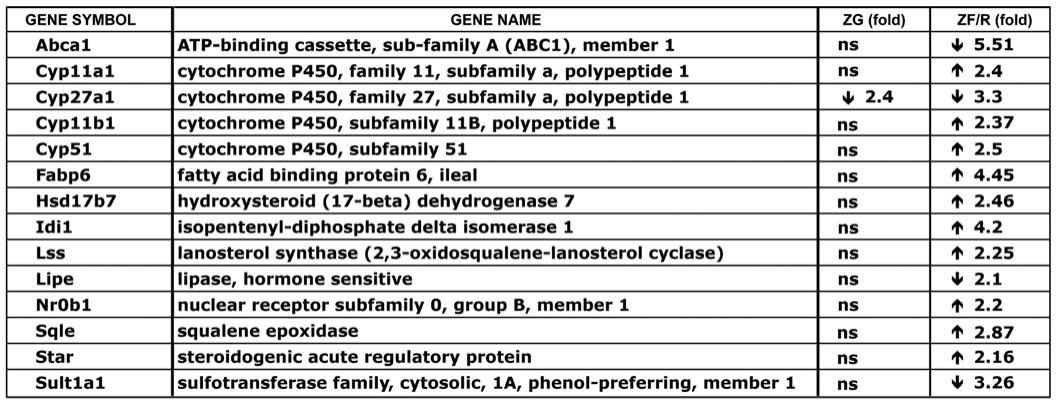
List of genes represented in ZF/R cluster 1, subcluster ‘steroid metabolic process’ (shown in Table II, DAVID analysis) and which are differentially expressed in the ZG and ZF/R of adrenal glands of adult female rats, in comparison to adult male rats. Downward facing arrowhead indicates a lower expression in females than in males; upward-facing arrowhead indicates a higher expression in females than in males; ns, differences statistically not significant; ZG, zona glomerulosa; ZF/R, zona fasciculata/reticularis.

Furthermore, analyses of ZF/R genes represented in cluster 1, subcluster ‘steroid metabolism process’ (from Table II) revealed numerous genes, the expression levels of which were different in the ZF/R (Fig. 6). Of these genes, the expression levels of only 4 of them [ATP-binding cassette, sub-family A (ABC1), member 1 (Abca1), cytochrome P450, family 27, subfamily A, polypeptide 1 (Cyp27a1), lipase, hormone-sensitive (Lipe) and Sult1a1] were lower in the females compared to the males. On the contrary, the remaining genes exhibited higher expression levels in the females [Cyp11a1, cytochrome P450, family 11, subfamily B, polypeptide 1 (Cyp11b1), cytochrome P450, family 51 (Cyp51), fatty acid binding protein 6, ileal (Fabp6), hydroxysteroid (17-beta) dehydrogenase 7 (Hsd17b7), isopentenyl-diphosphate delta isomerase 1 (Idi1), lanosterol synthase (2,3-oxidosqualene-lanosterol cyclase) (Lss), Nr0b1, squalene epoxidase (Sqle) and steroidogenic acute regulatory protein (Star)].

**Figure 7:**
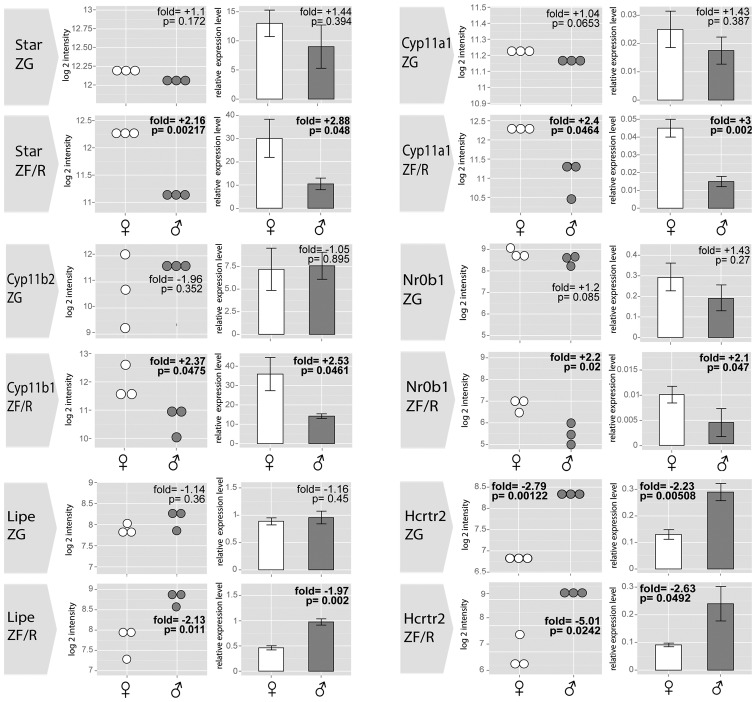
Results of RT-qPCR validation for selected genes with gender-specific differences in their expression levels in the ZG and ZF/R of adult male and female rats. Dot plots present data obtained from Affymetrix^®^ Rat Gene 1.1 ST Array, bar plots present the means ± SE from RT-qPCR. Eeach group n=3. Data are in comparison to male adrenal glands. Fold changes and p-values are shown. Star - steroid acute regulatory protein; Cyp11a1, cytochrome P450, family 11, subfamily A, polypeptide 1; Cyp11b2, cytochrome P450, family 11, subfamily B, polypeptide 2; Cyp11b1, cytochrome P450, family 11, subfamily B, polypeptide 1; Lipe, hormone sensitive lipase; Nr0b1, nuclear receptor subfamily 0, group B, member 1; Hcrtr2, hypocretin (orexin) receptor 2; ZG, zona glomerulosa; ZF/R, zona fasciculata/reticularis.

By means of RT-qPCR, we also validated the expression levels of selected genes with gender-specific differences in their expression levels in the ZG and/or ZF/R of adult male and female rat adrenal glands (Fig. 7). In all cases, the results of RT-qPCR confirmed the differences revealed by the Affymetrix^®^ Rat Gene 1.1 ST Array. The expression levels of Star, Cyp11a1 and Nr0b1 were notably higher in the ZF/R of the female rats, while no differences were observed in the ZG. No sex-related differences were observed in the expression levels of cytochrome P450, family 11, subfamily B, polypeptide 2 (Cyp11b2) in the ZG, while the expression level Cyp11b1 was notably higher in the female ZF/R. Lipe mRNA levels were notably higher in the male ZF/R, while in the ZG its expression was similar in both genders. Moreover, in both the ZG and ZF/R, the expression levels of hypocretin (orexin) receptor 2 (Hcrtr2) were notably lower in the female than in the male adrenal glands.

## Discussion

Global gene expression profiling allows for a simultaneous analysis of thousands of genes in a single sample. This powerful tool of molecular biology is widely used, among others, in studies on basic biology or in the diagnosis of various diseases. This method has also been used in studies on adrenal glands. In recent years increasing amounts of data linking gene expression with adrenal biology have been generated. In this area, the first reports were focused on gene profiles for steroidogenic enzymes in adrenocortical diseases, in particular in aldosterone-producing adenomas and other adrenocortical tumors (29–34). Gene profiling methods applied to freshly isolated adrenocortical cells or established cell lines (Y1, H295R human adrenocortical cells), allows the identification of numerous genes involved in the regulation of adrenocortical growth and functioning and provides novel data on intracellular pathways involved in the regulation of aldosterone and corticosterone secretion (13,35,36).

Transcriptional profiling has also been used in *in vivo* experiments. By means of this method, the circadian regulation of steroid hormone biosynthesis genes was examined in the rat adrenal gland (37). In knockout mice lacking Star, numerous up- and downregulated genes were identified by Ishii *et al* (38). Furthermore, recent studies on the regulation of the renin-angiotensin-aldosterone system (RAAS) in rat adrenal glands identified transcripts involved in RAAS activation (39). Transcriptional profiling has also been applied for the characterization of enucleation-induced adrenal regeneration in the rat (21–23).

From the above short survey, it appears that in *in vivo* experiments, gene profiling methods are used mainly for investigations of adrenal glands with experimentally modified function. Not considering studies on the circadian regulation of steroid hormone biosynthesis genes, to the best of our knowledge, only one study has been performed on the intact rat adrenal gland (14). The authors investigated differentially expressed transcripts in the adrenal ZG and ZF of the adult male rat. It appeared that in the ZG, 235 transcripts were upregulated by >2-fold compared to ZF and 231 transcripts were upregulated in the ZF (14). We performed a similar analysis of the adrenal glands of adult male and female rats. In our study, in both genders the number of differentially expressed transcripts in the adrenal ZG and ZF/R was notably higher than that previously reported for the ZG and ZF of male rats (14). The study by Nishimoto *et al* (14) identified 466 such transcripts, while the figures obtained in the present study amounted to 571 for male and 702 for female rats. These differences may be due to the various methods applied in two studies, as there are many methods gerenarally used (40–42). We used Affymetrix GeneChip^®^ WT Terminal Labeling and the Hybridization system, while Nishimoto *et al* applied the Illumina platform; we sampled fresh tissue (vs. frozen), used the RMA algorithm (vs. the percentile shift method) and our experiment was performed on Wistar rats (vs. the Sprague-Dawley strain) [we have also used Wistar rats in a previous study (43)]. Furthermore, the study by Nishimoto *et al* (14) obtained samples by means of a very precise laser-capture microdissection method, while our samples contained cells of both the zona fasciculata and reticularis (ZF/R). This means that our zona fasciculata cells were contaminated with the zona reticularis ones. Be as it may, our data also revealed notable differences in the number of differentially expressed transcripts in the adrenal glands of adult male and female rats, and in females this number was significantly higher.

It is well known that in the rat, the structure and function of the adrenal cortex exhibits sex-dependent differences (reviewed in refs. 4,5). The genetic background of these differences is little known, therefore we performed whole transcriptome analyses on the adrenal glands of mature male and female rats which allowed as to compare the expression levels of approximately 27,000 genes by applying microarray technology. In the adrenal cortex of the examined rats, in both the ZG and ZF/R, we identified 265 differentially expressed genes (male vs. female) and this figure is comparable to data previously reported for mice (269 genes regulated in the female adrenal compared to the male gland, and analyses were performed on the entire glands with Agilent array) (16). Our analysis revealed that the most pronounced sex-related differences in gene expression in the rat adrenal cortex were present in the ZF/R. In this compartment, the expression levels of 146 sex-regulated genes were lower in the female than in the male gland.

To describe the functions of differentially expressed genes in the male and female rat adrenal cortex, we performed the clustering of identified transcripts. By using DAVID tool, in the ZG, 3 annotations clusters were obtained and they were mainly related to ion transport and the response to endogenous stimuli. Unexpectedly, the expression levels of these genes were notably lower in the female rats. In the ZF/R, on the other hand, 5 annotation clusters with various numbers of subclusters were obtained. Of these, only in annotation cluster 1 (combining genes regulating steroid biosynthesis and metabolism) the numbers of upregulated transcripts in the female adrenal glands were higher than those in the male ZF/R. In the 4 remaining clusters, the numbers of downregulated transcripts in female adrenal glands were higher than those of the upregulated transcripts. These findings suggest the higher steroidogenic activity of the female than the male ZF/R and are in line with the findings of previous studies showing higher corticosterone secretion by the adrenal glands of female rats (4,5). Moreover, these observations are in accordance with those of previous studies, showing that stimulation of the highly-specialized function of adrenocortical cells (i.e., steroid hormone release) is always coupled with the inhibition or lowering of their basal biological processes, for instance the proliferation rate (44–46).

As revealed by DAVID analysis, the differentially expressed genes were significantly enriched in sets of genes involved in steroid hormone metabolism and their expression levels in the ZF/R of adult female rats were significantly higher than those in male adrenal glands. On the contrary, DAVID analysis did not reveal this group of transcripts in the ZG. Thus, the group of genes directly involved in steroid hormone synthesis expresses a sexually dimorphic pattern in the rat ZF/R, but not ZG. In this regard, the study by El Wakil *et al* (16) on sexual dimorphism of gene expression in the mouse adrenal gland revealed only 2 genes (Nr5a1 and Nr0b1) differentially expressed in the mouse, with higher expression levels in females. However, in their study none of the genes directly involved in steroid biosynthesis was found to be differentially expressed (16). We confirmed their findings on genes encoding known transcription factors and in the case of Nr0b1, we demonstrated that this difference was due to its higher expression in the ZF/R, but not in the ZG. Furthermore, the present study revealed that in the female ZF/R of the rat, there were higher expression levels of Cyp11a1, Cyp11b1, Fabp6, Hsd17b7, Idi1 and Star. These differences may be dependent on the species (rat vs. mouse) and the method of sampling, e.g., the separate anlaysis of the ZG and ZF/R in our study as opposed to the analysis of the entire glands in mice.

In contrast to ‘steroidogenic’ genes, in the female ZF/R, prevailing numbers of genes linked to cell fraction, oxidation/reduction processes and response to nutrients, as well as to extracellular stimuli or steroid hormone stimuli were found to be downregulated. Also this finding is rather unexpected, it seems to be scientifically justified (the negative interrelationship between highly specialized cell function and basic cell functions).

In present study, the expression levels of selected genes involved in the regulation of steroid biosynthesis was validated by RT-qPCR. As is known, cholesterol is the precursor for the entire adrenal steroidogenesis. Cholesterol may originate from different sources: i) *de novo* synthesis in the endoplasmic reticulum from acetyl CoA, ii) the mobilization of cholesteryl esters stored in lipid droplets through cholesteryl ester hydrolase, iii) plasma lipoprotein-derived cholesteryl esters obtained by either low-density lipoprotein (LDL) receptor-mediated endocytosis and/or scavenger receptor class B type I (SR-BI)-mediated selective uptake (47,48). Earlier data have demonstrated that the concentration of total lipids, total cholesterol, phospholipids and glycerides is similar in the adrenal glands of adult male and female rats; however their content, due to larger adrenal glands, is markedly higher in females (49). In this regard, Lipe is a major cholesterol hydrolase of the adrenal glands (50,51). The specific activity of this enzyme in 105,000 g supernatant of adrenal homogenates is higher in male than in female rats (52). In the present study, we demonstrated higher Lipe mRNA levels in the male ZF/R, while in the ZG the expression was similar in both genders. Thus, in the male ZF/R the higher activity of Lipe is accompanied by higher expression levels of the Lipe gene. Moreover, we demonstrated that the expression of the Lipe gene in the rat ZG was similar in both genders.

It has been well documented that the Star gene encodes a protein involved in the acute regulation of steroid hormone synthesis. This protein is responsible for the transport of cholesterol from the outer to the inner mitochondrial membrane (53). Earlier immunohistochemical and immunofluorescence studies have revealed the presence of Star protein in both the ZG and ZF/R of the rat adrenal cortex (54–57). In the mouse adrenal glands, Star mRNA levels (assessed by RT-PCR) have been reported be slightly higher in males than in females, although the differences were not statistically significant (58). In the present study, the mRNA expression levels of Star were notably higher in the ZF/R of female rats, while no differences were observed in the ZG. To the best of our knowledge, this is the first demonstration of specific sex-related differences in Star gene expression in the rat adrenal cortex.

Cyp11a1 encodes the P450scc enzyme (cholesterol 20–22 desmolase) that catalyzes the first and rate-limiting step of steroid biosynthesis (59,60). This enzyme is expressed in all adrenocortical zones (14,61). As previously reported, the rate of cholesterol transformation into pregnenolone (side chain cleavage activity) is markedly lower in male than in female rats (2,62,63). Moreover, the levels of adrenodoxin in decapsulated (i.e., devoid of ZG cells) adrenal glands have been reported to be lower in male than in female rats (64). In this study, we revealed the higher expression of the Cyp11a1 gene in the ZF/R of female rats. Again, as in the case of the Star gene, the mRNA levels of Cyp11a1 were comparable in the ZG of adult male and female rats. Thus, the previously reported higher rate of pregnenolone synthesis in the female rat adrenal cortex depends on the higher expression of the Cyp11a1 gene and the ZF/R compartment of the cortex is responsible for these differences.

We also validated the expression levels of 2 genes coding enzymes of the steroidogenic late pathway in the rat, Cyp11b2 (aldosterone synthase, responsible for aldosterone synthesis) and Cyp11b1 (steroid 11beta-hydroxylase, responsible for corticosterone synthesis). *In situ* hybridization and immunohistochemistry revealed the expression of aldosterone synthase in the rat ZG only (14,61,65). These results are in agreement with those of a previous study which demonstrated (by RT-PCR) high expression levels of Cyp11b2 in this zone (66). Our study confirmed these earlier observations. Moreover, we demonstrated that the expression levels of the Cyp11b2 gene were similar in the ZG of male and female rats. These data are in accordance with those of previous studies on the absence of sex-related differences in aldosterone synthesis in male and female rats (reviewed in Refs. 4,67). On the contrary, in the mouse adrenal glands, the mRNA levels of aldosterone synthase have been shown to be slightly higher in female than in male adrenal glands (58).

The Cyp11b1 gene encodes steroid 11beta-hydroxylase, which catalyzes the conversion of deoxycorticosterone to corticosterone. In the rat adrenal glands this enzyme is localized in the ZF/R cells (14,61). The activity of steroid 11beta-hydroxylase has been shown to be similar in adult male and female rats (68,69). However, the level of cytochrome P-450_11β_, in the rat adrenal mitochondria has been shown to be lower in male than in female rats (64). In the present study, we demonstrated that the Cyp11b1 gene expression levels were notably higher in the female ZF/R, while no differences were observed in the ZG of the adrenal cortex.

Nr0b1 encodes the Dax1 protein (dosage-sensitive sex reversal, adrenal hypoplasia critical region, on chromosome X, gene 1) which is responsible for the development and maintenance of the steroidogenic axis (70–72). Experimental data have suggested that the DAX1 protein is a negative regulator of steroidogenesis. The sexually dimorphic expression of Dax-1 (NR0B1) in the mouse adrenal cortex has been observed by RT-PCR, western blotting and immunohistochemistry (73). It should be mentioned that in the mouse adrenal glands, the mRNA levels of Dax1 were only slightly higher in female than in male adrenals (16,58). To the best of our knowledge, for the first time, we demonstrated that the expression levels of Nr0b1 were notably higher in the ZF/R of female rats, while no differences were observed in the ZG.

In a series of experiments, the group of Jöhren *et al* (74–76) demonstrated a notable sex-dependent expression of Hcrtr2 in the rat adrenal cortex. They demonstrated that Hcrtr2 was localized in the ZG and zona reticularis, with higher expression levels in male adrenal gland. We confirmed these earlier studies, as in our study, the expression levels of Hcrtr2 in the ZG and ZF/R were notably lower in the female than in the male adrenal glands. These results confirm the proper preparation of our tissue samples (ZG and ZF/R) for the analysis presented in this study.

In conclusion, to the best of our knowledge, the present study presents the first report of sex-related gene expression profiles in the adrenal cortex of adult rats. The number of differentially expressed transcripts in the adrenal ZG and ZF/R was notably higher in the female than in the male rats (702 vs. 571). The differentially expressed genes were significantly enriched in sets of genes involved in steroid hormone metabolism and their expression levels in the ZF/R of adult female rats were significantly higher than those in the male adrenal glands. In the female ZF/R, when compared with the male one, the prevailing numbers of genes linked to cell fraction, oxidation/reduction processes, response to nutrients and to extracellular stimuli or steroid hormone stimuli were downregulated.

## Figures and Tables

**Table I:** Primers used for the RT-qPCR validation of selected genes.

Gene symbol	Gene name	GenBank accession no.	Primer sequences (5′→3′)	Position	PCR product size (bp)
Star	Steroidogenic acute regulatory protein	NM_031558	CCTGAGCAAAGCGGTGTCATGCAAGTGGCTGGCGAACTCTA	745–764 911–931	187
Cyp11a1	Cytochrome P450, family 11, subfamily a, polypeptide 1	NM_017286	GATGACCTATTCCGCTTTGCGTTGGCCTGGATGTTCTTG	592–611 930–948	357
Cyp11b1	Cytochrome P450, family 11, subfamily b, polypeptide 1	NM_012537.3	TCATATCCGAGATGGTAGCACCTTCTGGGGATTAGCAACGA	863–883 1049–1068	206
Cyp11b2	Cytochrome P450, family 11, subfamily b, polypeptide 2	NM_012538.2	TGGCAGCACTAATAACTCAGGAAAAGCCACCAACAGGGTAG	875–895 1131–1150	276
Lipe	Hormone-sensitive lipase	NM_012859.1	GCCCTCCAAACAGAAACCCAAATCCATGCTGTGTGAGAA	967–985 1082–1101	135
Nr0b1	Nuclear receptor subfamily 0, group B, member 1	NM_053317.1	AGAGTACGCCTATCTGAAGATCGGTGTTGATGAATCTC	1141–1159 1321–1339	199
Hcrtr2	Hypocretin (orexin) receptor 2	NM_013074.1	GGCTTATCTCCAAATATTCCGTAACTCTGAACCACAGAAGAAGTTCC	782–806 828–850	69
Hprt	Hypoxanthine phosphoribosyltransferase	NM_012583	ATTTTGGGGCTGTACTGCTTGACAGTCAACGGGGGACATAAAAG	391–412 515–536	146

Gene symbol, gene names, GeneBank accession numbers, oligonucleotide sequences for sense and antisense primers, their position on mRNA and product size are shown. Hprt was used as a reference gene.

**Table II:** Functional annotation clustering report of differentially expressed genes in the ZG and ZF/R of adult male and female rats, as revealed by DAVID analysis (separate analyses for ZG and ZF/R).

Gene function	Up	Down

Zona gromelurosa	↓	↑
Annotation cluster 1/enrichment score: 3.73		
Regulation of calcium ion transport	3	1
Regulation of metal ion transport	3	1
Regulation of ion transport	3	1
Annotation cluster 2/enrichment score: 2.32		
Iron ion binding	4	1
Metal binding	8	1
Transition metal ion binding	8	2
Metal ion binding	10	2
Cation binding	10	2
Iron	3	1
Ion binding	10	2
Annotation cluster 3/enrichment score: 2.2		
Response to hormone stimuli	4	1
Response to endogenous stimuli	4	1
Response to steroid hormone stimuli	3	1

Zona fasciculata/reticularis		

Annotation cluster 1/enrichment score: 4.66		
Steroid metabolic process	4	10
Cholesterol metabolic process	3	6
Sterol metabolic process	3	6
Steroid biosynthetic process	0	8
Lipid biosynthetic process	3	11
Steroid biosynthesis	1	4
Annotation cluster 2/enrichment score: 3.75		
Microsome	8	6
Vesicular fraction	8	6
Insoluble fraction	15	7
Membrane fraction	14	7
Cell fraction	17	8
Annotation cluster 3/enrichment score: 2.99		
Oxidation reduction	14	9
Oxidoreductase	12	7
Monooxygenase	5	3
Electron carrier activity	7	4
Cytochrome P450, C terminal region	4	3
Cytochrome P450	4	3
Secondary metabolites biosynthesis, transport and catabolism	4	3
Iron ion binding	7	4
Iron	7	4
Cytochrome P450, conserved site	3	3
Chromoprotein	3	3
Metalloprotein	4	3
Cytochrome P450, E class, group I	4	1
Zona fasciculata/reticularis	↓	↑
Annotation cluster 4/enrichment score: 2.94		
Response to extracellular stimulus	7	7
Response to nutrient levels	6	6
Response to nutrients	4	6
Response to steroid hormone stimuli	6	5
Annotation cluster 5/enrichment score: 2.6		
Response to organic substance	17	11
Response to hormone stimuli	10	7
Response to endogenous stimuli	10	7
Response to steroid hormone stimuli	6	5
Response to glucocorticoid stimuli	4	3
Response to corticosteroid stimuli	4	3
Response to peptide hormone stimuli	5	4

With the selected cut-off value (p<0.05), tree annotation clusters were obtained in the ZG and 5 in the ZF/R. In the table clusters being ordered by the enrichment score, the higher the score, the more enriched the gene. Modified Fisher exact ‘p’ values and ‘p’ from Benjamini and Hochberg’s multiple testing correction are not shown. Up, upregulated; down, downregulated indicates higher or lower expression in relation to males. ZG, zona glomerulosa; ZF/R, zona fasciculata/reticularis.
